# Social Problems of the Day

**Published:** 1907-08-24

**Authors:** 


### SOCIAL PROBLEMS OF THE DAY.

### WILL UTOPIA EXCLUDE CHARITY?

### The Ethics of Charity.

Side by side with a rich growth of charitable effort there has come into being in recent years, and on the part of certain social reformers, a jealous dislike to what is commonly known as philanthropy. Never have charitable moneys been so wisely administered, and such good results been obtained, as at the very moment when the question begins to be insistently asked: "Is Charity a permanent and wholesome ingredient in civilised life, or is it merely a necessary evil? Ought it to find an entrance into the ideal community towards which progress tends?"

It will not do to obscure the question with any side issues as to the abuses of charity. It is charity in its wisest manifestations to which exception is taken. Nor can the dispute regarding the blessings conferred by charity be confined to the mere question of raising funds. Under one aspect charity does, it is true, consist in grants by one section of the community out of their surplus goods for the benefit of another section. But under another and far more important aspect, charity consists in personal service, in unremunerative altruistic labours, and it is this aspect, fully as much as the other, which the modern reformer holds in disfavour.

Mr. Wells, who is one of the most eloquent exponents of the "Down with Charity" creed, carries it sturdily into action in his new Utopia. Here everyone is self-sufficient, everyone prospers and develops, so far as Nature has lent him gifts and so far as it would be possible to prosper in a house with windows constructed after Mr. Wells' new patent, and never to be opened.

Nobody is occupied in doing good to anybody else. The inevitable drunkards, thieves, and murderers are shut away from the community in islands reserved for persons of similar tastes, where they are at liberty to cut each other's throats or to earn a precarious existence by picking each other's pockets. The theory that an evil-doer can have any one by ties of kindred or compassion who cares for his disgrace and is willing to lend him a helping hand, is not entertained. Altruism in this Utopia flows in a common-sense channel, and confines itself strictly to establishing wholesome habits in the normal citizen.

### The Real Points.

All this rests, of course, on the misleading assumption that mankind can be labelled definitely good, and bad, honest and dishonest, drunk and sober. It is comparatively easy both in morals and medicine to deal with the incurable. The crux of the whole matter in both spheres lies in the treatment of disease, and this is a matter in which the normal healthy citizen is not in the least interested. It will always be left for the altruist, whose sympathies have been developed till his own needs in his philosophy of life sink into the second place, to take thought for the weak, the suffering, and the morally defective. It is one of the healthiest signs of modern civilisation that this great field of altruistic labour, formerly abandoned almost without reserve to the private philanthropist, is being gradually reclaimed and tilled by the community. That the State should be called on to intervene in favour of the citizen who fails for any reason to be self-sufficient, points to the fact that the altruistic section of the community is in the ascendant. Moreover, this intervention is incontestable proof of the success of charitable effort, which must always in the first instance be purely experimental. So long as charity remains in the experimental stage, people are content to look on—tolerant, but not hopeful. When success is assured, the community awakes to the perception that operations of vital importance are being carried on by private persons, and not unnaturally insists on assuming the direction of events.

This course has been followed in many notable instances. It is but a short time back that education was the subject of purely private benevolence. The treatment of the sick, the care of the insane, the instruction of the blind and defective, the support of orphan children, the isolation of the fever-stricken, the housing of the poor, the encouragement of thrift—these are only a few of the directions in which private effort has come to be supplemented, and in some cases superseded, by public bodies. All this is perfectly normal in a community which advances in prosperity. Even that it should be accompanied by a sense of jealousy on the part of the philanthropist is not surprising. It is the great working population, every day rising to sounder perceptions of citizenship, which forms at once the field of philanthropic effort and the source from which municipal power is drawn. This working population, including roughly all who earn their own living, whether by commerce, profession, or hand labour, is exposed, irrespective of class, to perpetual reverses of fortune. Their independence rests on the quaking foundation of a continuance of the normal state of their affairs, and in proportion as they feel their collective strength and individual weakness, they revolt against resorting in their need to outsiders.

### State Philanthropy.

It is necessary, however, for a right understanding of the proper place of philanthropy in the State, to recognise that, if the community is to deal worthily with the distresses of the needy, it must consist of a large majority of workers earning at least rather more than sufficient for their own pressing needs. In a struggling and very poor community taxing itself for the relief of its necessitous members, the tendency is for power to fall into the hands of men who regard the misfortunes of others as evidence of weakness or criminality, because they can see but one side of the question, and that is, the hardship of being compelled to pause in their strenuous pursuit of independence to give a hand to the laggards. Such views are readily adopted by a population barely able by dint of incessant self-denial to keep their own families from want. The help granted is whittled down till it is just insufficient; and in the history of our own Poor-law administration may be traced the result. Even where the votes of the indigent keep in power a party strongly favourable to their claims, as in Paris at this moment, funds will be lacking just in proportion to the extent of the demands upon them. A population living for the most part on the border line of destitution, is unable to bear a taxation which would suffice to maintain in high efficiency the institutions and agencies demanded in the interests of the wholly destitute. And so we find in democratic Paris, where the rights of the poor are watched with jealous care, hospitals falling into decay for want of funds, and Public Assistance strained to breaking-point in its effort to cope merely with the worst cases of distress.

*To be continued.*

---

Mr. Gavin D. Muir, M.B., B.Ch., has been appointed Resident Medical Officer to the Royal Albert Hospital, Devonport.

